# New Copper(II) Coordination Compounds Assembled from Multifunctional Pyridine-Carboxylate Blocks: Synthesis, Structures, and Catalytic Activity in Cycloalkane Oxidation

**DOI:** 10.3390/molecules24010006

**Published:** 2018-12-20

**Authors:** Na Zhao, Yu Li, Jinzhong Gu, Tiago A. Fernandes, Marina V. Kirillova, Alexander M. Kirillov

**Affiliations:** 1 Foshan Research Center for Special Functional Building Materials and Their Green Preparation Technology, Guangdong Industry Polytechnic, Guangzhou 510300, China; 2012009035@gdip.edu.cn; 2 College of chemistry and Chemical Engineering, Lanzhou University, Lanzhou 730000, China; gujzh@lzu.edu.cn; 3 Centro de Química Estrutural, Instituto Superior Técnico, Universidade de Lisboa, Av. Rovisco Pais, 1049-001 Lisbon, Portugal; tiago.a.fernandes@tecnico.ulisboa.pt (T.A.F.); kirillova@tecnico.ulisboa.pt (M.V.K.); 4 Peoples’ Friendship University of Russia (RUDN University), Research Institute of Chemistry, 6 Miklukho-Maklaya st., Moscow 117198, Russia

**Keywords:** hydrothermal synthesis, crystal structure, coordination polymers, self-assembly, catalysis, alkanes, cyclohexane, oxidation reactions, hydrogen peroxide

## Abstract

Two new copper(II) coordination compounds, namely a 1D coordination polymer [Cu(µ-cpna)(phen)(H_2_O)]_n_ (**1**) and a discrete tetracopper(II) derivative [Cu(phen)_2_(H_2_O)]_2_[Cu_2_(µ-Hdppa)_2_(Hdppa)_2_] (**2**), were hydrothermally synthesized from copper(II) chloride as a metal source, 5-(4-carboxyphenoxy)nicotinic acid (H_2_cpna) or 5-(3,4-dicarboxylphenyl)picolinic acid (H_3_dppa) as a principal building block, and 1,10-phenanthroline (phen) as a crystallization mediator. Compounds **1** and **2** were isolated as air-stable microcrystalline solids and fully characterized by elemental and thermogravimetric analyses, IR spectroscopy, powder and single-crystal X-ray diffraction. In the solid state, the structure of **1** discloses the linear interdigitated 1D coordination polymer chains with the 2C1 topology. The crystal structure of an ionic derivative **2** shows that the mono- and dicopper(II) units are extended into the intricate 1D hydrogen-bonded chains with the SP 1-periodic net (4,4)(0,2) topology. Thermal stability and catalytic properties of **1** and **2** were also investigated. In fact, both Cu derivatives act as efficient homogeneous catalysts (catalyst precursors) for the mild oxidation of cycloalkanes by hydrogen peroxide to give the corresponding alcohols and ketones; the substrate scope and the effects of type and amount of acid promoter as well as bond-, regio-, and stereo-selectivity features were investigated.

## 1. Introduction

The design of new coordination polymers and derived materials has become a hot research topic in the fields of inorganic, coordination and materials chemistry, namely because of an almost unlimited structural diversity [1,2,3], unusual properties and promising applications of such compounds as functional materials. Examples of such applications range from the areas of luminescence [4,5,6] and molecular magnetism [7,8,9] to gas storage [10,11,12], sensing [13,14,15,16], and catalysis [17,18,19,20,21,22,23,24].

The structures and functional properties of target coordination polymers are influenced by a variety of factors that, among the reaction conditions, include the kind of metal nodes and organic building blocks [1,2,3,25,26,27,28,29,30]; the latter play a noteworthy structurally-driven role. A very common example of such building blocks concerns aromatic carboxylic acids, which attracted a considerable attention for assembling diverse coordination polymers [27,28,30,31,32,33].

**Scheme 1:**
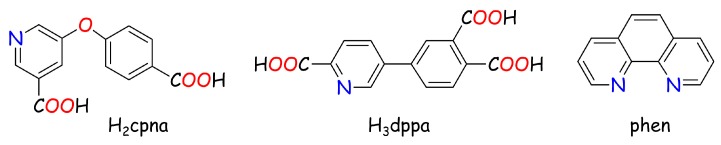
Structural formulae of H_2_cpna, H_3_dppa, and phen.

Following our general research interest toward the exploration of yet poorly investigated multicarboxylic acids for the synthesis of novel coordination polymers or metal-organic frameworks [31,32,33], in the present work we focused our attention on copper(II) ions as a metal source and 5-(4-carboxyphenoxy)nicotinic acid (H_2_cpna) or 5-(3,4-dicarboxylphenyl)picolinic acid (H_3_dppa) as main dicarboxylate or tricarboxylate building blocks, respectively (Scheme 1). The use of Cu(II) as metal nodes can be explained by their versatile coordination behavior and significance in bioinorganic chemistry [34,35] and oxidation catalysis [36,37,38,39]. On the other hand, the use of still little explored H_2_cpna and H_3_dppa can be justified by their multifunctionality (presence of N-pyridyl functionality and several COOH groups), good thermal stability and suitability for hydrothermal synthesis, as well as flexibility wherein pyridyl and phenyl rings can rotate around the C-O-C or C-C single bonds [40,41,42].

Hence, in the present study we probed the hydrothermal generation of coordination compounds from a multicomponent reaction mixture comprising of water as a solvent, copper(II) chloride as a metal source, H_2_cpna or H_3_dppa as a main building block, sodium hydroxide as a base (deprotonating agent), and phen (1,10-phenanthroline) as a crystallization mediator. As a result, two new products were isolated, i.e., [Cu(µ-cpna)(phen)(H_2_O)]_n_ (**1**) and [Cu(phen)_2_(H_2_O)]_2_[Cu_2_(µ-Hdppa)_2_(Hdppa)_2_] (**2**). Their full characterization, thermal behavior, structural and topological features, as well as catalytic properties toward the mild oxidation of cycloalkanes by H_2_O_2_ are reported herein.

## 2. Results and Discussion

### 2.1. Hydrothermal Synthesis

**Scheme 2:**
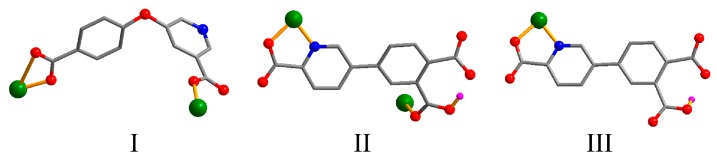
Coordination modes of µ-cpna^2−^ (mode I) and µ-Hdppa^2−^/Hdppa^2−^ (modes II, III) blocks in **1** and **2**.

Hydrothermal treatment of the aqueous mixtures composed of a copper(II) chloride, 5-(4-carboxyphenoxy)nicotinic acid (H_2_cpna) or 5-(3,4-dicarboxylphenyl)picolinic acid (H_3_dppa) as a principal building block, sodium hydroxide as a deprotonating agent, and 1,10-phenanthroline as a crystallization mediator gave rise to the generation of two novel coordination compounds, namely a 1D coordination polymer [Cu(µ-cpna)(phen)(H_2_O)]_n_ (**1**) and a discrete 0D 2Cu_1_ + Cu_2_ derivative [Cu(phen)_2_(H_2_O)]_2_[Cu_2_(µ-Hdppa)_2_(Hdppa)_2_] (**2**). Both products were isolated as stable microcrystalline solids and analyzed by standard solid-state characterization methods, including by single-crystal X-ray diffraction. The crystal structures of **1** and **2** are driven by pyridine-carboxylate blocks that adopt different coordination modes (Scheme 2).

### 2.2. Crystal Structure of [Cu(µ-cpna)(phen)(H_2_O)]_n_ (***1***)

**Figure 1:**
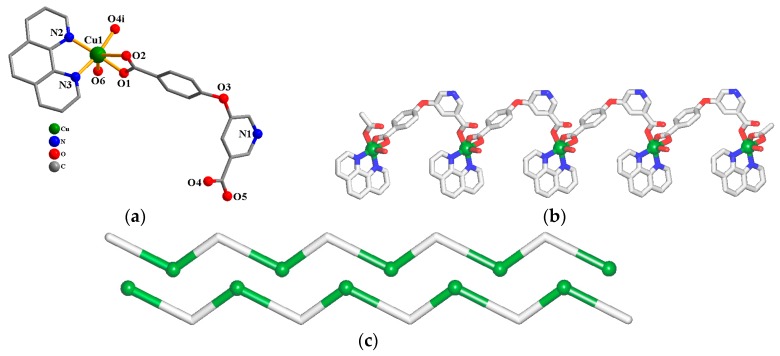
Structural fragments of **1**. (**a**) Coordination environment around the Cu(II) atom; H atoms are omitted for clarity. Symmetry code: i = *x*, *y*, *z*−1. (**b**) Linear 1D coordination polymer chain (view along the *a* axis). (**c**) Topological representation of two 1D chains showing a uninodal 2-connected underlying net with the 2C1 topology; color codes: 2-connected Cu nodes (green balls), centroids of 2-connected µ-cpna^2−^ linkers (gray).

In the solid state, compound **1** features a 1D coordination polymer structure that is driven by the µ-cpna^2−^ blocks (Figure 1). An asymmetric unit is composed of a Cu(II) center, a µ-cpna^2−^ block, a terminal phen ligand, and a coordinated H_2_O molecule. The Cu1 atom is six-coordinate and has a distorted octahedral {CuN_2_O_4_} environment, which comprises three carboxylate O donors from two µ-cpna^2−^ moieties, one H_2_O ligand, and two phen N donors (Figure 1a). The Cu-O [1.940(2)–2.932(3) Å] and Cu-N [2.031(3)–2.035(3) Å] distances are comparable to those in related Cu(II) derivatives [31,40,41,42]. In **1**, the cpna^2−^ moiety acts a μ-linker (mode I, Scheme 2) with its COO^−^ groups adopting a monodentate or bidentate modes; the nicotinate N atom remains uncoordinated. In μ-cpna^2−^, the dihedral angle between the two aromatic rings attains 78.26°, while the C-O_ether_-C angle reaches 117.96°. The μ-cpna^2−^ linkers multiply interconnect the adjacent Cu(II) centers to generate a 1D linear chain structure (Figure 1b); the adjacent chains are interdigitated. Topologically, such chains are composed of the 2-connected Cu1 nodes and the µ-cpna^2−^ linkers (Figure 1c), and are classified as a uninodal 2-connected underlying net with the 2C1 topology.

### 2.3. Crystal Structure of [Cu(phen)_2_(H_2_O)]_2_[Cu_2_(µ-Hdppa)_2_(Hdppa)_2_] (***2***)

**Figure 2:**
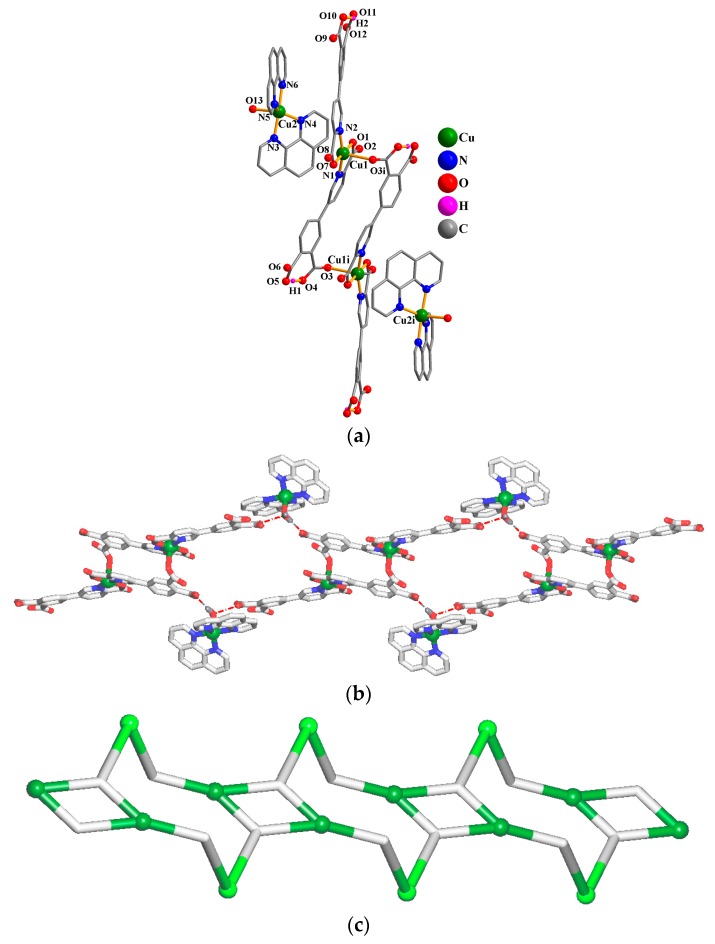
Structural fragments of **2**. (**a**) Coordination environment around the Cu(II) atoms; H atoms are omitted for clarity except those in COOH groups. Symmetry code: i = −*x*, −*y*, −*z*. (**b**) 1D H-bonded chain (view along the *c* axis); H bonds are shown as dotted lines. (**c**) Topological representation of a decorated 1D chain showing a uninodal 3-connected underlying net with the SP 1-periodic net (4,4)(0,2) topology (view along the *a* axis); color codes: 3-connected Cu1 nodes (dark green balls), centroids of 3-connected μ-Hdppa^2−^ nodes or 2-connected Hdppa^2−^ linkers (gray), centroids of 2-connected [Cu(H_2_O)(phen)_2_]^2+^ linkers (green balls).

The crystal structure of **2** (Figure 2a) is composed of two [Cu(phen)_2_(H_2_O)]^2+^ cations (two monocopper(II) blocks, 2Cu_1_) and a [Cu_2_(µ-Hdppa)_2_(Hdppa)_2_]^4−^ anion (a dicopper(II) block, Cu_2_). In the cation, the Cu2 atom adopts a distorted trigonal bipyramidal {CuN_4_O} geometry filled by four N atoms from two phen moieties and one H_2_O ligand. In the anion, the Cu1 center is also five-coordinate and reveals a distorted trigonal bipyramidal {CuN_2_O_3_} environment, which is taken by two carboxylate O and one N atom from two different µ-Hdppa^2−^ blocks, as well as one carboxylate O and one N atom from the terminal Hdppa^2−^ ligand. The Cu-O and Cu-N bonds span in the 1.944(3)–2.406(4) and 1.963(4)–2.060(4) Å range, respectively; these are within the normal values for related Cu(II) derivatives [31,40,41,42]. In **2**, the Hdppa^2−^ blocks act as a terminal monodentate ligand (mode III, Scheme 2) or as a µ-linker (mode II), having the corresponding dihedral angles of 9.64° or 19.24° between pyridyl and phenyl rings. In the anion, two µ-Hdppa^2−^ moieties link two adjacent Cu1 atoms to form a Cu_2_ unit with a Cu⋯Cu separation of 9.375(2) Å (Figure 2a). The dicopper(II) [Cu_2_(µ-Hdppa)_2_(µ-Hdppa)_2_]^4−^ anions and monocopper [Cu(H_2_O)(phen)_2_]^2+^ cations are arranged into the intricate 1D H-bonded double chains (Figure 2b). After simplification, such 1D chains can be topologically defined as a uninodal 3-connected underlying net (Figure 2c) with the SP 1-periodic net (4,4)(0,2) topology and the point symbol of (4^2^.6).

### 2.4. Thermogravimetric and Powder X-ray Diffraction Analysis

Thermal behavior and stability of compounds **1** and **2** were studied by thermogravimetric analysis (TGA) in the 20–800 °C temperature range under N_2_ atmosphere (Figure S1). TGA curve of **1** shows a release of one coordinated water molecule between 111 and 162 °C (exptl, 3.3%; calcd, 3.5%); a dehydrated solid remains stable on further heating up to 225 °C. In **2**, a weight loss in the 112–156 °C range corresponds to a removal of two H_2_O ligands (exptl, 1.9%; calcd, 1.7%) and the dehydrated material keeps its integrity on heating up to 210 °C.

Microcrystalline samples of **1** and **2** were also subjected to PXRD (powder X-ray diffraction) study. PXRD patterns of the bulk products are given in Figures S2 and S3. The experimental results well match the diffractograms simulated from the single-crystal X-ray diffraction data, thus confirming a phase purity of the bulk samples of **1** and **2**.

### 2.5. Mild Catalytic Oxidation of Cycloalkanes

**Scheme 3:**
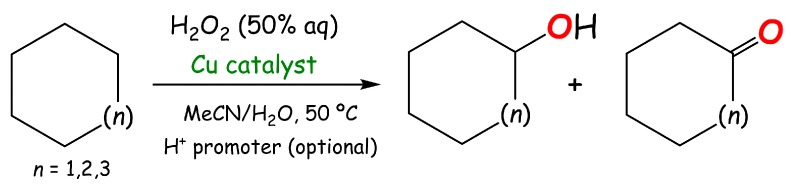
Cu-catalyzed oxidation of cycloalkanes to cyclic alcohols and ketones.

Compounds **1** and **2** were tested as homogeneous catalysts (catalyst precursors) in the mild oxidation of C_6_–C_8_ cycloalkanes to the corresponding alcohols and ketones (Scheme 3). Reactions were typically run in acetonitrile medium at 50 °C in air, using 50% aqueous hydrogen peroxide as an oxidant, and in the presence (optional) of an acid promoter. Trifluoroacetic acid (TFA), HNO_3_, and HCl were tested as typical promoters [36,37,38,39]. It should be mentioned that both the coordination polymer **1** and an ionic complex **2** dissolve in the catalytic reaction medium and produce homogeneous catalytically active species. Cyclohexane was used as a model substrate for detailed catalytic studies due to the industrial significance of its oxidation products, cyclohexanone and cyclohexanol, that are intermediates in the production of nylon [43,44]. In fact, an industrial process for the oxidation of cyclohexane (DuPont) also operates with a homogeneous metal carboxylate catalyst (cobalt naphthenate), requires harsher reaction conditions, and shows a maximum C_6_H_12_ conversion of only ~5–10% [43,44]. Herein, a higher catalytic activity was achieved in the presence of **2**, resulting in up to 25% of the total product yield. Both catalysts (catalyst precursors) show a similar trend toward substrate reactivity: C_6_H_12_ < C_8_H_16_ < C_7_H_14_.

**Figure 3:**
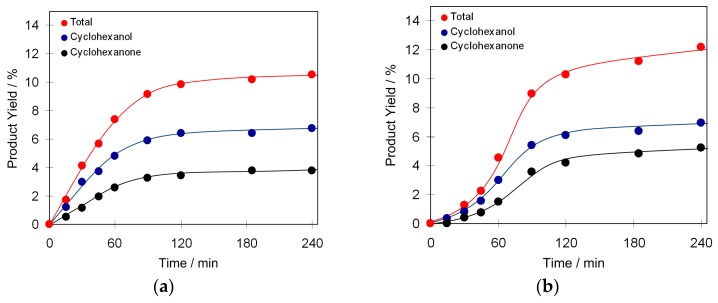
Kinetic curves of product accumulation in the cyclohexane oxidation by H_2_O_2_ catalyzed by **1** (**a**) and **2** (**b**) in the presence of TFA promoter. Reaction conditions: Cu catalyst or catalyst precursor (5.0 (**1**) or 2.5 (**2**) μmol), TFA (0.05 mmol), C_6_H_12_ (1.0 mmol), H_2_O_2_ (5.0 mmol), CH_3_CN (up to 2.5 mL of the total volume), 50 °C.

Compounds **1** and **2** catalyze the oxidation of cyclohexane and show a comparable level of activity, resulting in ~10–12% of the total yield (Figure 3) with cyclohexanol being formed in higher amount in comparison with cyclohexanone (~1.5–2:1 molar ratio). However, there is a difference in kinetic curves. In the case of **1**, there is a smooth accumulation of the products up to 120 min, whereupon no yield increase is observed. In contrast, in the cyclohexane oxidation catalyzed by **2**, there is a lag period of ~45 min when the reaction is accelerating, showing then a maximum reaction rate up to 120 min. The lag period can be associated with a lower solubility of **2** in the reaction medium.

**Figure 4:**
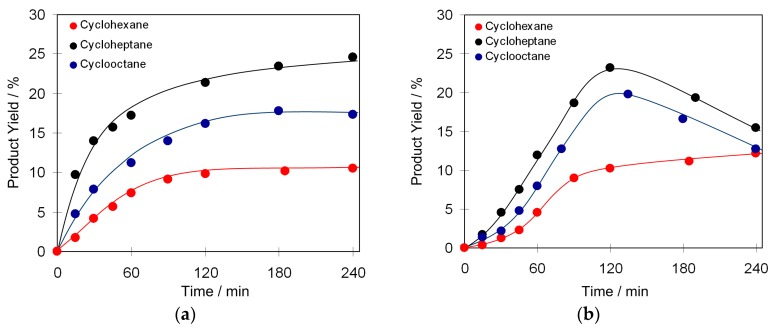
Mild oxidation of cycloalkanes (C_6_–C_8_) to the corresponding alcohols and ketones (total yield) by H_2_O_2_ catalyzed by **1** (**a**) and **2** (**b**). Reaction conditions are those of Figure 3.

Oxidations of cycloheptane and cyclooctane proceed more efficiently than that of cyclohexane for both **1** and **2** (Figure 4). Cycloheptane is the most reactive substrate resulting in the yields (total of cycloheptanol and cycloheptanone) of ~25 and 23% for **1** and **2**, respectively. Oxidation of cyclooctane leads to 17–20% total product yields. The corresponding kinetic curves of products accumulation in C_7_H_14_ and C_8_H_16_ oxidations are different for compounds **1** and **2**. In the case of **1**, reactions proceed up to 120 min and then the yields practically do not change (no overoxidation was observed). In contrast, in the oxidations catalyzed by **2** and after an achievement of the maximum value (at 120 min), the yield drop was detected due to an overoxidation. Besides, the lag period, detected for C_6_H_12_ oxidation in the presence of **2**, is less pronounced for cyclooctane and cycloheptane.

**Figure 5:**
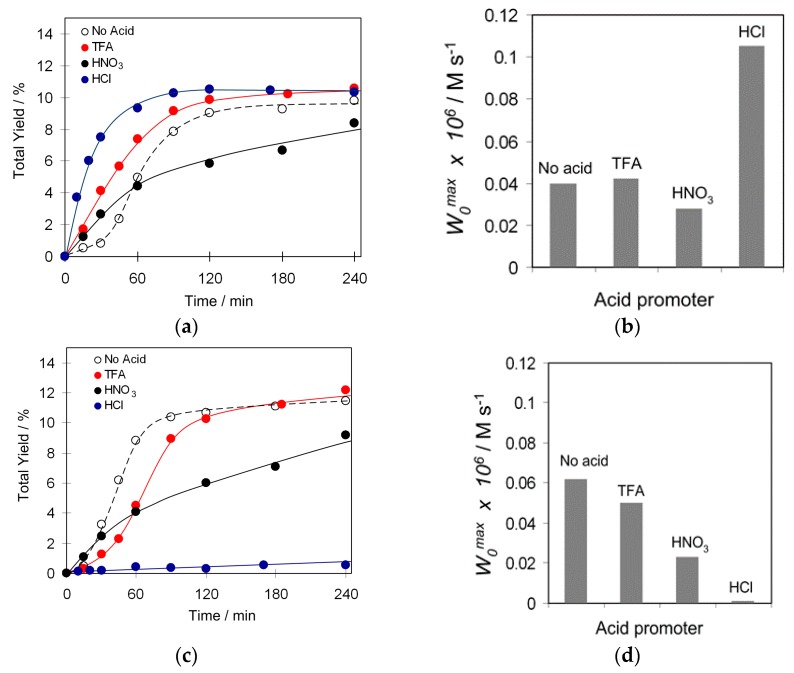
Effect of the acid promoter (**a**,**c**) on the total yield of cyclohexanol and cyclohexanone and (**b**,**d**) maximum initial reaction rate (*W*_0_) in the cyclohexane oxidation with H_2_O_2_ catalyzed by **1** (**a**,**b**) and **2** (**c**,**d**). Reaction conditions are those of Figure 3.

In contrast to other Cu-based catalytic systems, the compounds **1** and **2** do not require any acid promoter to catalyze the oxidation of cycloalkanes (Figure 5). However, in the case of **1**, the presence of an acid promoter (HCl, TFA or HNO_3_) in a low amount (acid-to-catalyst molar ratio of 10:1) leads to the acceleration of the reaction and removes a minor lag period (Figure 5a). Overall efficiency of the system is higher in the presence of HCl and TFA, wherein the reactions proceed faster and result in slightly superior total yields. Although HNO_3_ is capable of removing a lag period, the oxidation of cyclohexane catalyzed by the **1**/HNO_3_ system is slower and less efficient. The maximum initial reaction rate was observed in the presence of HCl (Figure 5b).

A different behavior is noticed for catalyst **2** in the presence of acid promoter (Figure 5c,d). Interestingly, the highest activity (total yield and *W*_0_*^max^*) is attained in the absence of any promoter. The presence of HCl results in the full suppression of catalytic activity of **2**, whereas the addition of TFA leads to a slight deceleration of the reaction and extends an existing lag period; the same product yield was observed as in the absence of acid. An addition of HNO_3_ results in lowering the product yield and the reaction rate.

**Figure 6:**
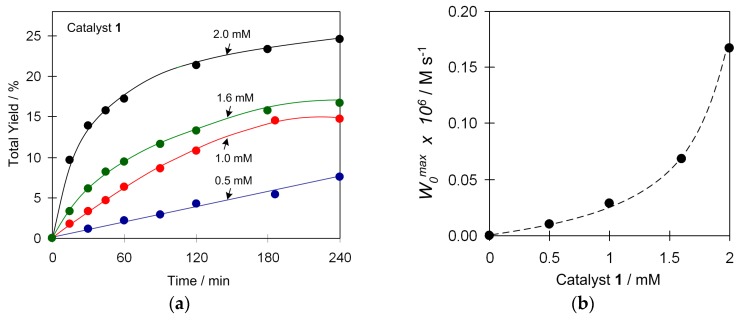
Effect of the amount of **1** on (**a**) the total yield of cycloheptanol and cycloheptanone and (**b**) the maximum initial reaction rate (*W*_0_) in the cycloheptane oxidation with the **1**/H_2_O_2_ system. Reaction conditions: **1** (0.5–2.0 mM), acid promoter (TFA, 0.05 mmol), C_7_H_14_ (1.0 mmol), H_2_O_2_ (5.0 mmol), 50 °C, CH_3_CN (up to 2.5 mL total volume).

Since the highest activity was observed for the cycloheptane oxidation catalyzed by **1**, we studied the effect of the amount of **1** on the total yield of the products (cycloheptanol and cycloheptanone) and the maximum initial reaction rate (Figure 6). Both the total yield and *W*_0_*^max^* are growing by increasing the catalyst precursor amount from 0.5 to 2.0 mM, revealing the *W*_0_*^max^* dependence on the concentration of **1** with an order > 1. It possibly indicates that more than one Cu containing moiety participates in the rate limiting step of the reaction.

It should be noted that the coordination polymer **1** is not intact in the course of catalytic tests and undergoes a partial disaggregation upon dissolution and in the presence of oxidant and/or acid promoter to give a homogeneous catalytically active species. To get more information on the type of species present in solution, we investigated the model aqueous solutions of **1** and **1**/H_2_O_2_ by ESI-MS(+), using the conditions typical to those of catalytic tests. The following main fragments can be observed in the MS(+) spectrum of **1**: [Cu(µ-cpna)(phen)_2_ + H]^+^ (*m*/*z* 681), [Cu(µ-cpna)(phen) + H]^+^ (*m*/*z* 501), and [Cu(phen)_2_]^+^ (*m*/*z* 423). Besides, an intense peak of the molecular ion (*m*/*z* 501) is also detected after addition of H_2_O_2_. Such data suggest a fragmentation (can also be ESI-induced) of coordination polymer **1** upon dissolution to generate a series of monocopper species. These, along with the related cationic [Cu(phen)_2_(H_2_O)]^2+^ and anionic [Cu_2_(Hdppa)_4_]^4−^ blocks of **2**, most likely represent the homogeneous catalytically active species in the present oxidation reactions.

The observed efficiency of the present catalytic systems is comparable to other copper-based systems applied in the mild oxidation of cycloalkanes [20,36,37,38,39]. However, the majority of these catalytic systems also requires the use of an acid promoter in contrast with the catalytic behavior of **1** and **2**. These are capable of catalyzing the oxidation of cycloalkane in the absence of added acid, what can be associated with the presence of pyridine-carboxylate ligands in the structures of **1** and **2** [45].

### 2.6. Regio- and Bond Selectivity Investigation and Proposed Mechanism

In order to obtain additional information on the nature of the oxidizing species, we tested the oxidation of branched alkanes in the presence of both catalytic systems (Table 1). In the oxidation of methylcyclohexane, the normalized bond selectivity parameters 1°:2°:3° of 1:4:11 (**1**) and 1:5:17 (**2**) suggest that the tertiary C atom is oxidized with some preference over the secondary C atoms. Similar behavior is observed in the adamantane oxidation that shows the 2°:3° parameter of 1:3.2 (determined as the ratio of the formed tertiary and secondary alcohol isomers). Oxidation of *n*-heptane proceeds without specific preference to any secondary C atom of the hydrocarbon chain, revealing the C(1):C(2):C(3):C(4) value of 1:6:6:7 and 1:5:5:7 in the presence of **1** and **2**, respectively. *Trans*-dimethylcyclohexane is oxidized with low stereoselectivity (*trans*/*cis* ratio of the isomer products is 0.9). The above selectivity parameters are indicative of a powerful and rather indiscriminate oxidizing species such as hydroxyl radicals [20,36,37,38,39,46].

Summarizing these results, we can propose that the general reaction mechanism involves the following steps [20,44,45,47,48]. H_2_O_2_ interacts with a copper catalyst (catalyst precursor) causing the formation of oxo/peroxo-copper intermediates via the coordination of H_2_O_2_ followed by the elimination of HO^•^ radicals. Then, the hydroxyl radicals abstract H atoms from a cycloalkane producing the alkyl radicals R^•^, which further react with O_2_ (from air) and result in the ROO^•^ radicals. These can be transformed into cycloalkyl hydroperoxides ROOH as primary intermediate products. Then, cycloalkyl hydroperoxides decompose (conceivably by Cu-catalyzed processes in the course of the reaction) to furnish the corresponding alcohols and ketones as final products.

## 3. Experimental

### 3.1. Materials and Physical Measurements

All chemicals were of analytical reagent grade and used as received. H_2_cpna and H_3_dppa were obtained from Jinan Henghua Sci. & Tec. Co., Ltd (Jinan, China). IR spectra were recorded on a Bruker EQUINOX 55 spectrometer (Bruker Corporation, Billerica, MA, USA) using KBr pellets. Elemental (C, H, N) analyses were run on an Elementar Vario EL elemental analyzer (Elementar, Langenselbold, Germany). Thermogravimetric analyses (TGA) were performed under N_2_ atmosphere on a LINSEIS STA PT1600 thermal analyzer (Linseis Messgeräte GmbH, Selb, Germary) with a heating rate of 10 °C/min. Powder X-ray diffraction patterns (PXRD) were measured on microcrystalline samples using a Rigaku-D/MAX 2400 diffractometer (Cu-Kα radiation; *λ* = 1.54060 Å) (Rigaku Corporation, Tokyo, Japan). ESI-MS(+) measurements were performed on a LCQ Fleet mass spectrometer with an ESI source (Varian, Inc., Palo Alto, CA, USA).

### 3.2. Synthesis of [Cu(µ-cpna)(phen)(H_2_O)]_n_ (***1***)

A mixture of CuCl_2_·2H_2_O (34.1 mg, 0.2 mmol), H_2_cpna (51.8 mg, 0.2 mmol), phen (39.6 mg, 0.2 mmol), NaOH (16.0 mg, 0.4 mmol), and H_2_O (10 mL) was stirred at room temperature for 15 min. Then, it was sealed in a 25 mL Teflon-lined stainless steel vessel and heated at 120 °C for 3 days, followed by cooling to room temperature at a rate of 10 °C/h. Green block-shaped crystals were isolated manually, washed with distilled H_2_O, and dried in air to give compound **1**. Yield: 65% (based on H_2_cpna). Calcd for C_25_H_17_CuN_3_O_6_: C 57.86, H 3.30, N 8.10%. Found: C 58.01, H 3.32, N 8.06%. IR (KBr, cm^−1^): 3383 w, 3067 w, 2927 w, 1610 s, 1558 m, 1517 w, 1496 m, 1428 m, 1367 s, 1340 m, 1288 w, 1242 m, 1206 w, 1164 m, 1097 w, 1024 w, 1009 w, 962 w, 905 w, 848 m, 780 m, 724 m, 693 w, 651 w, 614 w.

### 3.3. Synthesis of [Cu(phen)_2_(H_2_O)]_2_[Cu_2_(µ-Hdppa)_2_(Hdppa)_2_] (***2***)

Compound **2** was prepared following the procedure described for **1** but using H_3_dppa (57.4 mg, 0.2 mmol) instead of H_2_cpna. Blue block-shaped crystals were isolated manually, washed with distilled H_2_O, and dried in air to give **2**. Yield: 55% (based on H_3_dppa). Calcd for C_104_H_64_Cu_4_N_12_O_26_: C 58.05, H 3.00, N 7.81%. Found: C 57.84, H 3.02, N 7.75%. IR (KBr, cm^−1^): 3378 w, 3062 w, 1685 w, 1622 s, 1591 m, 1516 m, 1428 m, 1368 s, 1253 w, 1166 w, 1100 w, 1039 w, 964 w, 913 w, 848 m, 819 w, 772 m, 725 m, 698 w, 644 w, 535 w.

### 3.4. X-ray Crystallography and Topological Analysis

Single-crystal X-ray data for **1** and **2** were collected on a Bruker APEX-II CCD diffractometer, using a graphite-monochromated Mo *K*_α_ radiation (*λ* = 0.71073 Å). Semiempirical absorption corrections were applied using the SADABS program. Crystal structures were determined using direct methods and refined by full-matrix least-squares on *F*^2^ with the SHELXS-2015 and SHELXL-2015 programs [49]. All the non-H atoms were refined anisotropically by full-matrix least-squares methods on *F*^2^. All the H atoms (except those of H_2_O/COOH) were placed in calculated positions with fixed isotropic thermal parameters, and included in structure factor calculations at the final stage of full-matrix least-squares refinement. Hydrogen atoms of H_2_O/COOH moieties were located by difference maps and constrained to ride on their parent oxygen atoms. Crystal data for **1** and **2** are given in Table 2. Selected bond lengths and hydrogen bonding details are given in Tables S1 and S2, respectively (Supplementary Material). CCDC-1877525 and 1877526 for **1** and **2** contain the supplementary crystallographic data.

Topological analysis of coordination (**1**) or H-bonded (**2**) networks was performed following the concept of the simplified underlying net [50]. Underlying nets were generated by: (i) eliminating terminal ligands and contracting bridging ligands to their centroids in **1** or (ii) contracting µ-Hdppa^2−^/Hdppa^2−^ ligands and [Cu(H_2_O)(phen)_2_]^2+^ blocks to their centroids in **2**. Connectivity of nodes and linkers was maintained via coordination (in **1**) or both coordination and hydrogen bonds (in **2**). Only strong D–H⋯A hydrogen bonds were considered, wherein the H⋯A < 2.50 Å, D⋯A < 3.50 Å, and ∠(D−H⋯A) > 120°; D and A stand for donor and acceptor atoms, respectively [51].

### 3.5. Mild Oxidation of Cycloalkanes

Cycloalkane oxidation reactions were typically performed in air atmosphere in thermostated glass reactors equipped with a condenser under vigorous stirring at 50 °C under atmospheric pressure and using MeCN as solvent (up to 2.5 mL total volume). These conditions of temperature and pressure are considered as rather mild in the field of alkane oxidation [20,39,40]. In a typical experiment, copper(II) catalyst or catalyst precursor (5.0 µmol for **1** or 2.5 µmol for **2**), acid promoter (optional, 0.05 mmol) and gas chromatography (GC) internal standard (MeNO_2_, 25 µL) were introduced into MeCN solution, followed by an addition of alkane substrate (1 mmol). Reaction started by adding hydrogen peroxide (50% in H_2_O, 5 mmol) in one portion. The oxidation reactions were monitored by withdrawing small aliquots of the reaction mixture after different periods of time, which were treated with PPh_3_ for the reduction of remaining H_2_O_2_ and alkyl hydroperoxides that are typically formed as primary products in alkane oxidations [52,53]. The samples were then analyzed by GC using nitromethane as an internal standard. The formation of alkyl hydroperoxides as primary intermediate products was also confirmed by GC analyses of the reaction mixtures before and after the treatment with PPh_3_ (Shul’pin’s method) [52,53]. Attribution of peaks was made by comparison with chromatograms of authentic samples. Blank tests confirmed that alkane oxidations do not proceed in the absence of copper catalyst.

## 4. Conclusions

In this work, we studied the hydrothermal generation of copper(II) coordination compounds from a three-component system comprising Cu(II) chloride—pyridine carboxylic acid—1,10-phenanthroline. Two new products **1** and **2** were generated depending on the type of the main multicarboxylate building block. Their solid-state structures revealed different types of metal-organic or H-bonded 1D chains, which also represent rare examples of coordination compounds derived from H_2_cpna and H_3_dppa as principal building blocks. The present study also widens the application of hydrothermal synthetic protocols and use of water as a benign solvent for the generation of novel coordination compounds.

Besides, copper(II) compounds **1** and **2** act as homogeneous catalysts (catalyst precursors) for the mild oxidation of C_6_–C_8_ cycloalkanes (cyclohexane, cycloheptane, and cyclooctane) by H_2_O_2_ to give a mixture of the respective cyclic alcohols and ketones, resulting in up to 25% total product yields based on cycloheptane. Such yields are considered rather high in the area of alkane functionalization, especially taking into account a high inertness of these saturated hydrocarbons and the mild reaction conditions applied (e.g., 50 °C temperature, atmospheric pressure, aqueous H_2_O_2_ oxidant). Additional studies on the heterogenization of the obtained compounds on a solid support will be pursued aiming at the development of recoverable heterogeneous catalysts.

Further research on exploring the present types of multifunctional pyridine-carboxylic acids for the hydrothermal synthesis of coordination polymers or metal-organic frameworks and search for their applications in oxidation catalysis are currently in progress in our laboratories.

## Figures and Tables

**Table 1:** Different selectivity parameters in the mild oxidation of methylcyclohexane, adamantine, *n*-heptane, and *trans*-dimethylcyclohexane catalyzed by **1** and **2**. ^a^

Selectivity Parameter	1	2
*Bond selectivity*		
1°:2°:3° (methylcyclohexane) ^b^	1:4:11	1:5:17
2°:3° (adamantane) ^b^	1:3.3	1:3.2
*Regioselectivity*		
C(1):C(2):C(3):C(4) (*n*-heptane) ^c^	1:6:6:7	1:5:5:7
*Stereoselectivity*		
*trans*/*cis* (*trans*-dimethylcyclohexane) ^d^	0.9	0.9

^a^ Reaction conditions: alkane (1.0 mmol), catalyst or catalyst precursor (5 (**1**) or 2.5 (**2**) µmol), TFA (0.05 mmol), H_2_O_2_ (50% aq., 5.0 mmol), MeCN (up to 2.5 mL total volume), 50 °C, 2 h. All parameters were calculated based on the ratios of isomeric alcohols. The parameters were normalized, i.e., recalculated taking into account the number of H atoms at each carbon atom; ^b^ Parameters 1°:2°:3° and 2°:3° are relative normalized reactivities of the H atoms at primary (only for methylcyclohexane), secondary, and tertiary carbon atoms of methylcyclohexane and adamantane, respectively; these are determined as the ratio of the formed primary, secondary, and tertiary alcohol isomers; ^c^ Parameters C(1):C(2):C(3):C(4) are the relative reactivities of hydrogen atoms at carbons 1, 2, 3 and 4 of the *n*-heptane chain; ^d^ Parameter *trans*/*cis* is determined as the ratio of the formed tertiary alcohol isomers with mutual *trans* and *cis* orientation of the methyl groups.

**Table 2:** Crystal data for compounds **1** and **2**.

Compound.	1	2
Chemical formula	C_25_H_17_CuN_3_O_6_	C_104_H_64_Cu_4_N_12_O_26_
Molecular weight	518.95	2151.87
Crystal system	Monoclinic	Monoclinic
Space group	*P*2_1_/*c*	*P*2_1_/*c*
*a*/Å	8.7501(4)	17.9277(7)
*b*/Å	23.0910(7)	14.6238(8)
*c*/Å	11.0499(4)	16.4405(7)
*α*/(°)	90	90
*β*/(°)	110.506(4)	98.168(4)
*γ*/(°)	90	90
*V*/Å^3^	2091.15(14)	4266.5(3)
*Z*	4	4
*F*(000)	1060	2192
Crystal size/mm	0.25 × 0.23 × 0.22	0.25 × 0.23 × 0.21
*θ* range for data collection	3.298–28.624	3.322–28.586
Limiting indices	−8 ≤ *h* ≤ 11, −15 ≤ *k* ≤ 29, −14 ≤ *l* ≤ 13	−24 ≤ *h* ≤ 18, −16 ≤ *k* ≤ 19, −20 ≤ *l* ≤ 22
Reflections collected/unique (*R*_int_)	9097/4733 (0.0400)	19416/9809 (0.0568)
*D*_c_*/*(Mg·cm^−3^)	1.648	1.675
*μ*/mm^−1^	1.096	1.080
Data/restraints/parameters	4733/0/316	9809/0/660
Goodness-of-fit on *F*^2^	1.051	1.035
Final *R* indices[(*I* ≥ 2σ(*I*))] *R*_1_, w*R*_2_	0.0503, 0.0943	0.0627, 0.1080
R indices (all data) *R*_1_, w*R*_2_	0.0828, 0.1113	0.1345, 0.1446
Largest diff. peak and hole/(e·Å^−3^)	0.402 and −0.414	0.443 and −0.556

## Supplementary Materials

The following data are available online. Figure S1: TGA curves, Figures S2 and S3: PXRD patterns, Tables S1 and S2: selected bonding and H-bonding parameters for compounds 1 and 2, Schemes S1 and S2: additional catalysis schemes (bond selectivity study).
