# Supplementary material for: New Copper(II) Coordination Compounds Assembled from Multifunctional Pyridine-Carboxylate Blocks: Synthesis, Structures, and Catalytic Activity in Cycloalkane Oxidation
Source: Molecules. 2018 Dec 20;24(1):6. doi: 10.3390/molecules24010006 (PMC6337171; doi:10.3390/molecules24010006)
Supplement: Supplementary file 1 [file molecules-24-00006-s001.pdf]

Supplementary Material

New Copper(II)-Organic Architectures Assembled from Multifunctional Pyridine-Carboxylate Blocks: Hydrothermal Generation, Structural Features, and Mild Catalytic Oxidation of Cycloalkanes

Na Zhao[1], Yu Li [1,*], Jinzhong Gu [2], Tiago A. Fernandes [3], Marina V. Kirillova [3] and Alexander M. Kirillov [3,4*]

[1] Foshan Research Center for Special Functional Building Materials and Its Green Preparation Technology, Guangdong Industry Polytechnic, Guangzhou 510300, China; 853759800@qq.com (N.Z.)

[2] College of chemistry and Chemical Engineering, Lanzhou University, Lanzhou 730000, China; gujzh@lzu.edu.cn (J.G.)

[3] Centro de Química Estrutural, Instituto Superior Técnico, Universidade de Lisboa, Av. Rovisco Pais, 1049-001, Lisbon, Portugal; tiago.a.fernandes@tecnico.ulisboa.pt (T.A.F), kirillova@tecnico.ulisboa.pt (M.V.K.)

[4] Peoples' Friendship University of Russia (RUDN University), Research Institute of Chemistry, 6 Miklukho-Maklaya st., Moscow, 117198, Russian Federation

* Correspondence: liyuletter@163.com (Y.L.); kirillov@tecnico.ulisboa.pt (A.M.K.); Tel.: +86-20-61230629

Supplementary material contains:

Figure S1 TGA curves for compounds **1** and **2**.

Figure S2 Powder X-ray diffraction patterns of **1**.

Figure S3 Powder X-ray diffraction patterns of **2**.

Table S1 Selected bond lengths (Å) and bond angles (°) for compounds **1** and **2**.

Table S2 Hydrogen bonds in crystal packing [Å, °] for compounds **1** and **2**.

Scheme S1: Cu-catalyzed oxidation of methylcyclohexane (bond selectivity study).

Scheme S2: Cu-catalyzed oxidation of adamantane (bond selectivity study).

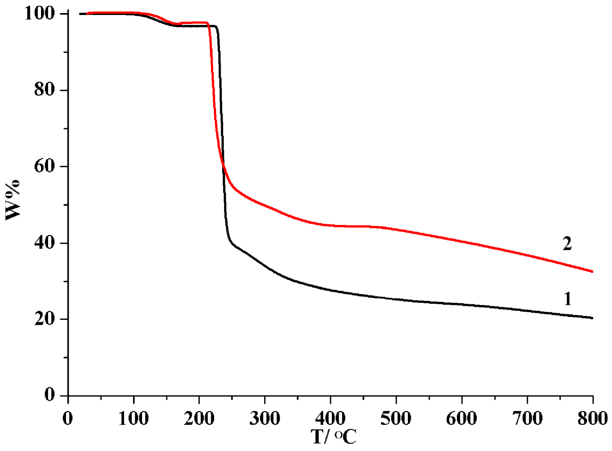

Figure S1 TGA curves for compounds **1** and **2**.

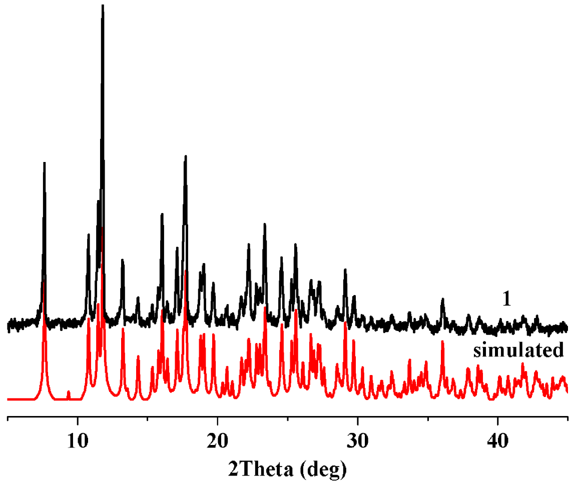

Figure S2 The PXRD patterns of compound **1** at room temperature.

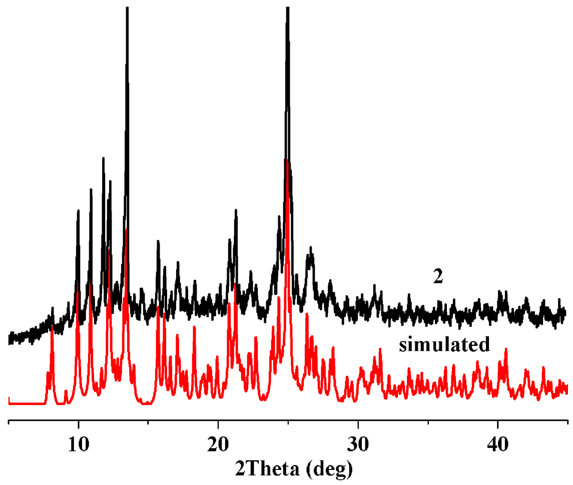

. **Figure S3** The PXRD patterns of compound **1** at room temperature.

Table S1 Selected bond lengths (Å) and bond angles (°) for compounds **1** and **2**.

		**1**			
Cu(1)–O(1)	1.944(2)	Cu(1)–O(2)	2.932(3)	Cu(1)–O(4)i	1.940(2)
Cu(1)–O(6)	2.309(3)	Cu(1)–N(2)	2.034(3)	Cu(1)–N(3)	2.031(3)
O(1)–Cu(1)–O(2)	49.08(10)	O(1)–Cu(1)–O(6)	89.01(9)	O(1)–Cu(1)–N(2)	175.23(10)
O(1)–Cu(1)–N(3)	94.34(10)	O(1)–Cu(1)–O(4)i	95.28(10)	O(2)–Cu(1)–O(6)	137.17(8)
O(2)–Cu(1)–N(2)	129.37(10)	O(2)–Cu(1)–N(3)	93.20(11)	O(2)–Cu(1)–O(4)i	87.61(10)
O(6)–Cu(1)–N(2)	92.76(10)	O(6)–Cu(1)–N(3)	94.62(10)	O(4)i–Cu(1)–O(6)	92.85(9)
N(2)–Cu(1)–N(3)	81.11(11)	O(4)i–Cu(1)–N(2)	89.07(10)	O(4)i–Cu(1)–N(3)	167.92(10)
		**2**			
Cu(1)–O(1)	1.946(3)	Cu(1)–O(3)i	2.406(4)	Cu(1)–O(7)	1.944(3)
Cu(1)–N(1)	1.971(4)	Cu(1)–N(2)	1.963(4)	Cu(2)–O(13)	2.151 (3)
Cu(2)–N(3)	1.998(4)	Cu(2)–N(4)	2.042(4)	Cu(2)–N(5)	2.061(4)
Cu(2)–N(6)	1.993(4)				
O(1)–Cu(1)–O(7)	178.89(1)	O(1)–Cu(1)–N(1)	83.31(1)	O(1)–Cu(1)–N(2)	96.56(1)
O(1)–Cu(1)–O(3)i	91.74(1)	O(7)–Cu(1)–N(1)	96.67(1)	O(7)–Cu(1)–N(2)	83.78(1)
O(3)i–Cu(1)–O(7)	87.18(1)	N(1)–Cu(1)–N(2)	163.44(1)	O(3)i–Cu(1)–N(1)	100.97(1)
O(3)i–Cu(1)–N(2)	95.59(1)	N(3)–Cu(2)–N(5)	100.04(1)	N(3)–Cu(2)–N(6)	175.80(1)
O(13)–Cu(2)–N(3)	87.37(1)	O(13)–Cu(2)–N(4)	120.12(1)	O(13)–Cu(2)–N(5)	104.77(1)
O(13)–Cu(2)–N(6)	88.61(1)	N(3)–Cu(2)–N(4)	82.08(1)	N(4)–Cu(2)–N(6)	98.93(1)
N(5)–Cu(2)–N(6)	82.16(1)	N(4)–Cu(2)–N(5)	135.10(1)		

Symmetry codes: (**1**) i: $x, y, z - 1$; (**2**) i: $-x, -y, -z$.

Table S2 Hydrogen bonds in crystal packing [Å, °] for compounds **1** and **2**.

Compound	D–H···A	*d*(D–H)	*d*(H···A)	*d*(D···A)	∠DHA	Symmetry code
**1**	O(6)–H(1W)···O(5)	0.88	2.02	2.702	133.8	$x, y, z - 1$
	O(6)–H(2W)···O(1)	0.85	1.99	2.841	179.4	$-x + 1, -y + 1, -z + 1$
**2**	O(4)–H(1)···O(5)	1.06	1.37	2.395	159.7	
	O(10)–H(2)···O(11)	0.95	1.73	2.421	126.7	
	O(13)–H(1W)···O(12)	0.85	1.85	2.697	179.3	$-x + 1, -y + 1, -z + 1$
	O(13)–H(2W)···O(6)	0.86	1.87	2.713	166.8	$-x, -y + 1, -z$

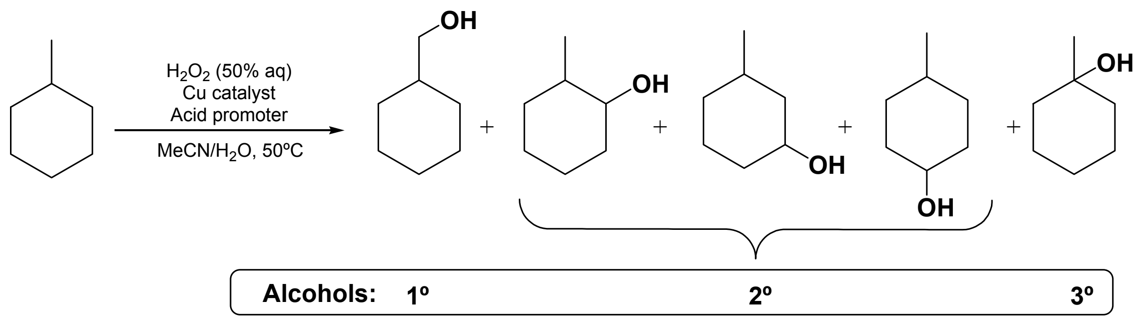

Scheme S1: Cu-catalyzed oxidation of methylcyclohexane (bond selectivity study).

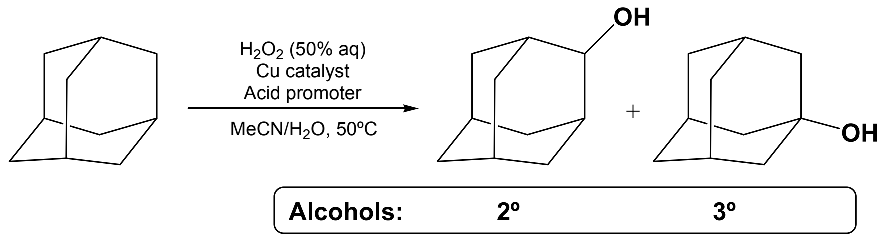

Scheme S2: Cu-catalyzed oxidation of adamantane (bond selectivity study).
